# A cross-sectional study of the availability and pharmacist’s knowledge of nano-pharmaceutical drugs in Palestinian hospitals

**DOI:** 10.1186/s12913-018-3060-7

**Published:** 2018-04-05

**Authors:** Mohyeddin Assali, Ali Shakaa, Sabaa Abu-Hejleh, Reham Abu-Omar, Nareman Karajeh, Nawal Ajory, Saed Zyoud, Waleed Sweileh

**Affiliations:** 1 Department of Pharmacy, Faculty of Medicine and Health Sciences, An-Najah National University, Nablus, Palestine; 2 Department of Biomedical Sciences, Faculty of Medicine and Health Sciences, An-Najah National University, Nablus, Palestine

**Keywords:** Pharmaceutical nanotechnology, Nano-pharmaceutical drugs, Availability, Knowledge, Pharmaceutical nano-formulation, Palestinian hospitals

## Abstract

**Background:**

Nanomedicine is the medical application of nanomaterials that may have an infinite size with the range less than 100 nm. This science has provided solutions to many of the current limitations in the diagnosis and treatment of diseases. Therefore, the pharmacist’s knowledge and awareness of nano-pharmaceutical drugs will increase their availability in the market, and will improve the patient’s compliance to their drug therapy. This study aimed to determine the availability of nano-pharmaceutical drugs in Palestinian hospitals and evaluate the extent of pharmacist’s knowledge about them.

**Methods:**

A cross-sectional study design questionnaire was used to determine the availability of nano-pharmaceutical drugs based on the database of the ministry of health in the Palestinian hospitals (governmental, private and non- governmental organizations). Moreover, the knowledge of these nano-pharmaceutical drugs among pharmacists working in Palestinian hospitals was assessed based on developed questionnaire from the literature of the pharmaceutical formulations and nano-formulations. The variables were analyzed using Statistical Package for Social Sciences (SPSS 22).

**Result:**

Fifty six pharmacists from 27 hospitals in the West bank completed the survey. The results regarding the availability of nano-pharmaceutical drugs indicated only eight available in hospitals with a frequency range 0–39.3%. Moreover, pharmacist’s knowledge in the pharmaceutical formulations was better than that in nano-formulations.

**Conclusions:**

The availability of nano-pharmaceutical drugs in Palestinian hospitals was not adequate due to the lack of various nano-pharmaceutical drugs. The knowledge among pharmacists regarding nano-pharmaceutical drugs should be improved by providing courses in nanomedicine during the undergraduate pharmacy programs.

**Electronic supplementary material:**

The online version of this article (10.1186/s12913-018-3060-7) contains supplementary material, which is available to authorized users.

## Background

Nanotechnology is a new science that focuses on the understanding and control of matter at the nanoscale, with dimensions between approximately 1 and 100 nm, which enable novel applications in medicine, drug delivery, electronics, biomaterials, energy storage, agriculture and many others [1, 2]. Current advances in nanotechnology have led to the development of a new field of science called nanomedicine or pharmaceutical nanotechnology, which includes many applications of nanomaterials and nanodevices for diagnostic and therapeutic purposes [3]. Nanomedicine aims to improve the therapeutic index of some conventional drugs, by enhancing their water solubility, and provide specific targeting to decrease their side effects [4].

Nanomedicine is the science of diagnosing, treating, preventing diseases, and improving human health, using molecular tools and molecular knowledge of the human body with highly specific medical intervention at the molecular scale for reducing diseases or repairing damaged tissues based on nanotechnology, which focuses on the level of molecule and atom [5]. Research priorities in nanomedicine include drug delivery and targeting of pharmaceutical, therapeutic and diagnostic agents [6]. These involve the identification of the precise targets (cells and receptors) related to specific clinical conditions and the choice of the appropriate nanocarriers to achieve the required responses while minimizing the side effects [7, 8].

There are now many types of nanomaterials that have been utilized in the field of nanomedicine and have shown interesting applications such as liposomes [9, 10], nano-emulsions [11], polymeric nanoparticles [12, 13], carbon nanotubes [14], gold nanoparticles [15] and others. Moreover, there are more than 100 drugs approved by the FDA as nano-pharmaceutical drugs available in different dosage forms and for variety applications [16]. For example, Doxil® (Doxorubicin) was the first- FDA approved nano-pharmaceutical drug in 1995. It has a marked prolonged circulation time due to the insertion of polyethylene glycol chain so it is a peglated nano-liposome that escape from host immune cells, this property allows the drug to be effective for a longer time with less side effects [17]. Ambisome® (Amphotericin B) is a small unilamellar liposome preparation (45–80 nm) containing amphotericin B in the bilayer. The reduced toxicity and elevated peak plasma level of Ambisome® compared with amphotericin B is achieved without loss of the broad-spectrum antifungal activity of amphotericin B [18]. Peg-Intron® (interferon alpha 2B) is a peglated polymer nanoparticle of interferon alpha 2B used in chronic hepatitis C with high anticancer effect [19]. Due to the importance of nanomedicine, various reviewers have discussed the significant value of nanomedicine in the medical field [20–24]. Kirtane et al. have summarized the importance of nano-pharmaceutical drugs in the field of cancer therapy [23]. Pourmand et al. have reviewed the application of nanomedicine in emergency medicine and the opportunity to diagnose and treat life-threatening diseases in a shorter period of time [24]. From the previous mentioned reviews, we can observe the importance of nanotechnology in the field of medicine and the extent of the applications that can be obtained from this new science and technology of research. Therefore, it is necessary to increase the awareness and the knowledge of the medical practitioners such as pharmacists, doctors and biomedical research scientists of the importance of pharmaceutical nanotechnology and the already existing drugs that have many advantages in the cure and treatment of various diseases.

However, the drug’s formulary management status in Palestinian hospitals is limited and the hospital pharmacy is not a well defined section of pharmacy practice [25]. Moreover, Zaid et al. has reported recently the lack of pharmaceutical compounding in Palestine. The reasons for these limitations are the lack of regulations and the insufficient training and equipments for practicing pharmaceutical compounding [26]. Therefore, the main sources of drugs in Palestine is the local pharmaceutical manufacturers and some international imported drugs. The local manufacturers are generic companies that lack the research and development of new drugs [25]. Since nanomedicine is a new field of pharmaceutical formulations, the local pharmaceutical companies don’t provide any nano-pharmaceutical drugs and they are only available from international imported pharmaceutical warehouse.

Therefore, this study is aim to assess the knowledge of pharmacists in nano-pharmaceutical drugs and the availability of nano-pharmaceutical drugs in the Palestinian hospitals. Herein, the targeted group of the study is the pharmacists, since they are the primary source who deal with the pharmaceutical formulations and know the availability of these nano-drugs in the market. To our knowledge, this study will be first of its kind locally and internationally.

## Methods

### Study design

A cross sectional study design questionnaire was used that involved descriptive and comparative analysis. The list of the supposed available nano-pharmaceutical drugs has been obtained from the ministry of health website (http://pharmacy.moh.ps/index/RegisteredProducts/Language/en), the Palestinian medical index (PMI®) and the Israeli medical index (medic®).

### Study area & sample size

The data collection was conducted from September to November 2015. A list of hospitals was obtained from the Ministry of Health website (http://pharmacy.moh.ps/index/RegisteredProducts/Language/en). Twenty seven hospitals are the total number of the available hospitals in the West Bank exclude Gaza strips and Jerusalem hospitals, eleven of them governmental, eight private and eight NGOs. The estimated sample size is 56 pharmacists which is the total number of the working pharmacists in the studied hospitals.

### Data collection

Data collection was achieved by face-to-face interviews with the pharmacists in their work place. Our structured questionnaire was prepared in Arabic and involved three sections. Additional file 1 contains the English questionnaire and is divided into the following sections: the first one is the socio-demographic section which contains questions as: profession/gender, experience (years), graduation university (local /International), education level (bachelor / master), type of Hospital (private / NGO /government).

The second section contained four conventional pharmaceutical formulations questions and other four pharmaceutical nano-formulations questions which were obtained from previous studies [13, 27–34] with Crohn’s bach alpha = 0.6 in order to measure the pharmacist’s knowledge.

Finally the third section which asked about the availability of nano-pharmaceutical drugs in Palestinian hospitals and the frequency of consumption.

### Ethical approval

Before initiation of this study we received approval from the Palestinian Ministry of Health and Institutional Review Board (IRB) at An-Najah National University who also approved the verbal consent procedure. Verbal agreement from the pharmacists was also obtained.

### Statistical analysis

The statistical package for social sciences program version 22 (IBM-SPSS) was used to enter and analyze our data. Variables were tested for normality using the Kolmogorov-Smirnov test. Continuous data were expressed as median (interquartile range). All variables were nonparametric, categorical data have expressed as frequencies (percentage) and a *p* value of less than 0.05 was considered statistically significant. The Mann-Whitney U test or Kruskal Wallis test was performed as appropriate.

## Results

### Socio-demographic

Fifty six pharmacists from 27 hospitals in the West bank completed the survey. Table 1 shows socio-demographic characteristics of the study participants. By analyzing the collected data, 64.3% of the sample were females, the majority of the sample were graduates from a local university (71.4%). 87.5% of the sample had a bachelor degree. The majority had working experience in the hospital pharmacy of 0–5 years with a percentage of 44.6%. 51.8% of the hospitals in the sample were governmental, 28.6% private and 19.6% were Non-Governmental Organizations.

**Table 1:** Socio -demographic characteristics of the study participants

Variable	Frequency (%) *N* = 56
Gender	
Male	20 (35.7)
Female	36 (64.3)
Type of Hospital	
Governmental	29 (51.8)
Private	16 (28.6)
NGOs	11 (19.6)
Education level	
Bachelor	49 (87.5)
Master	7 (12.5)
Graduation university	
Local Universities	40 (71.4)
International universities	16 (28.6)
Experience (years)-hospital pharmacy-	
0–5	25 (44.6)
5–10	14 (25.0)
> 10	17 (30.4)

### Knowledge among pharmacists of pharmaceutical formulations and nano-formulations

The knowledge section was divided into eight questions to measure the knowledge of hospital pharmacists in the field of conventional pharmaceutical formulations and nano- formulations. The questions were collected from the literature as shown in Table 2. Four of the questions are related to conventional pharmaceutical formulations which are enteric coated, sublingual, corticosteroids inhaler and suppositories as presented in Table 3 (questions # 1, 3, 5 and 7 respectively). The other four questions are related to pharmaceutical nano-formulations that include liposomes, nano-emulsions, polymeric nanoparticles and nanocrystals suspensions as shown in Table 3 (questions # 2, 4, 6 and 8 respectively). Table 3 shows the asked questions, the frequency and the percentage of the pharmacist’s answers. It can be observed that the correct pharmacist’s answers were much higher in the field of pharmaceutical formulations in comparison to nano-formulations. The percentage of correct answers in the case of pharmaceutical formulations were 87.5%, 92.9%, 76.0%, 78.6% in questions # 1, 3,5 and 7 respectively. On the other hand, the percentage of correct answers in the case of pharmaceutical nano-formulations were 55.4%, 39.3%, 42.9%, 44.6% in questions # 2, 4, 6 and 8 respectively.

**Table 2:** The asked questions with the correct answers and the frequency of each answer

No.	Questions	Frequency (%) *N* = 56
1)	Enteric coated [34] Choose the most characteristic feature of this pharmaceutical dosage form:	
	a) Reduce the number of doses needed from the drug and achieve a slow/sustained release of the drug.	2 (3.6)
	^a^ *b) Aid in the protection of the drug from stomach acidity.*	*49 (87.5)*
	c) Used when the patient is susceptible to vomiting.	0 (0.0)
	d) I don’t know.	5 (8.9)
2)	Liposomes [32] Choose themost characteristic feature of this pharmaceutical dosage form:	
	a) Used when the patient is susceptible to vomiting.	1 (1.8)
	b) Aid in the protection of the drug from stomach acidity.	2 (3.6)
	^a^ *c) Reduce the side effects of the drug and improve its pharmacokinetic profile.*	*31 (55.4)*
	d) I don’t know.	22 (39.3)
3)	Sublingual [30] Choose the most characteristic feature of this pharmaceutical dosage form:	
	a) Reduce the number of doses needed from the drug and achieve a slow/sustained release of the drug.	0 (0.0)
	b) Protect the drug from degradation or hydrolysis.	2 (3.6)
	^a^ *c) Fast drug absorption and protect the drug from hepatic metabolism.*	*52 (92.9)*
	d) I don’t know.	2 (3.6)
4)	Nano emulsions [30] Choose the most characteristic feature of this pharmaceutical dosage form:	
	^a^ *a) Reduce the number of doses needed from the drug and achieve a slow/sustained release of the drug.*	*22 (39.3)*
	b)Aid in the protection of the drug from stomach acidity.	0 (0.0)
	c)Used when the patient is susceptible to vomiting.	4 (7.1)
	d) I don’t know.	30 (53.6)
5)	Corticosteroid Inhaler [29] Choose the most characteristic feature of this pharmaceutical dosage form:	
	a) Allow the delivery of low water solubility drugs and permit theslow/sustained release of the drug.	9 (16.1)
	^a^ *b) The drug goes directly to the lungs which reduce the required dose and its side effects.*	*43 (76.8)*
	c)Used when the patient is susceptible to vomiting.	0 (0.0)
	d) I don’t know.	4 (7.1)
6)	Polymer Nanoparticles [31] Choose the most characteristic feature of this pharmaceutical dosage form:	
	^a^ *a) Protect the drug from degradation or hydrolysis.*	*24 (42.9)*
	b) The drug goes directly to the lungs which reduces the required dose and its side effects*.*	7 (12.5)
	c)Used when the patient is susceptible to vomiting.	1 (1.8)
	d) I don’t know.	24 (42.9)
7)	Suppositories [27] Choose the most characteristic feature of this pharmaceutical dosage form:	
	a) Protect the drug from degradation or hydrolysis.	7 (12.5)
	b) Reduce the number of doses needed from the drug and achieve a slow/sustained release of the drug.	2 (3.6)
	^a^ *c) Used when the patient is susceptible to vomiting.*	*44 (78.6)*
	d) I don’t know.	3 (5.4)
8)	Nano crystal dispersion [28] Choose the most characteristic feature of this pharmaceutical dosage form:	
	a)Aid in the protection of the drug from stomach acidity.	4 (7.1)
	^a^ *b) Allow the delivery of low water solubility drugs and permit the slow/sustained release of the drug.*	*25 (44.6)*
	c) Used when the patient is susceptible to vomiting.	0 (0.0)
	d) I don’t know.	27 (48.2)

^a^means the right answer

**Table 3:** Variable affect on pharmacist’s knowledge

Variable	Frequency (%) *N* = 56	Median pharmaceutical formulation score [interquartile range]	*p*-value	Median pharmaceutical Nano-formulation score [interquartile range]	*p*-value	*P-*value between the median of pharmacutical formulation and nanoformulation
Gender						
Male	20 (35.7)	4.0 [3.25–4.00]	0.157^a^	2.5 [1.25–3.00]	0.159 ^a^	< 0.001^a^
Female	36 (64.3)	3.0 [3.00–4.00]		1.0 [0.25–3.00]		< 0.001^a^
Type of Hospital						
Governmental	29 (51.8)	3.0 [2.50–4.00]	0.515^b^	2.0 [1.00–3.00]	0.579 ^b^	< 0.001^a^
Private	16 (28.6)	4.0 [3.00–4.00]		1.5 [1.00–3.00]		< 0.001^a^
NGOs	11 (19.6)	4.0 [2.00–4.00]		1.0 [0.00–3.00]		0.007^a^
Education level						
Bachelor	49 (87.5)	4.0 [3.00–4.00]	0.92 ^a^	2.0 [0.50–3.00]	0.092 ^a^	< 0.001^a^
Master	7 (12.5)	4.0 [3.00–4.00]		3.0 [1.00–3.00]		0.117^a^
Graduation university						
Local Universities	40 (71.4)	3.0 [2.25–4.00]	0.018 ^a^	2.0 [0.25–3.00]	0.390 ^a^	< 0.001^a^
International Universities	16 (28.6)	4.0 [4.00–4.00]		2.0 [1.00–3.00]		< 0.001^a^
Experience (years)						
0–5	25 (44.6)	3.0 [2.50–4.00]	0.139 ^b^	2.0 [1.00–3.00]	0.920 ^b^	< 0.001^a^
5–10	14 (25.0)	4.0 [3.75–4.00]		2.0 [1.00–3.00]		< 0.001^a^
> 10	17 (30.4)	3.5 [2.50–4.00]		1.0 [0.00–3.00]		0.001^a^

^a^ Statistical significance of differences calculated using the Mann-Whitney U test

^b^ Statistical significance of differences calculated using the Kruskal Wallis test

*P*-value > 0.05 is insignificant value

### Variables effects on pharmacist’s knowledge

Various variables such as gender, type of hospital, educational level, graduation university and the experience years were studied in order to determine their effects on pharmacist’s knowledge. As it can be observed in Table 3, in all variables the pharmacist’s knowledge in the pharmaceutical nano-formulations was less than their knowledge in the conventional pharmaceutical formulations. Moreover, there is no difference in knowledge between male and female, the type of hospital, educational level or the year of experience as the *p* value in all cases was insignificance. However, there was a significant difference in the pharmacist’s knowledge between the local and international graduation universities regarding their knowledge in the conventional pharmaceutical formulations, but there was no difference in their knowledge in the nano-formulations. These results confirm that the pharmaceutical nanotechnology is a new science and neither the experience nor the graduation university have improved the pharmacist’s knowledge in this new field.

Moreover, a comparison study was conducted between all the variables and the knowledge of conventional pharmaceutical formulations and nano-formulations. As shown in Table 3, the variables (gender -male and female-, type of hospital -governmental, private and NGOs-, education level -bachelor-, graduation university -local universities and international universities-, years of experience − 0-5, 5–10, > 10-) have a *p*-value less than 0.05 which indicates that there is significant differences in knowledge between conventional pharmaceutical formulations and nano-formulations, which confirms the obtained results. On the other hand, the master education level had a *p*-value of 0.117 which indicates that there is no significant difference in knowledge. This could be due to that the pharmacists with a master degree could have studied advance courses in pharmaceutical formulation which have included nano-formulation as a part of these courses.

### Availability of nano-pharmaceutical drugs in Palestinian hospitals

A list of the available registered nano-pharmaceutical drugs in the Palestinian ministry of health (MoH), Palestinian medical index (PMI®) and the Israeli medical index (medic®) was developed according to the FDA approved nano-pharmaceutical drugs as shown in Table 4. There are only eight nano-pharmaceutical drugs available in the Palestinian hospitals from a total of twelve registered nano-pharmaceutical drugs. The availability of nano-drugs varied substantially according to hospital type. However, these differences did not reach statistical significance except in the case of Emend® (Aprepitant) and Swiss Relief® (Diclofinac sodium). Moreover, the frequency of the availability of the nano-pharmaceutical drugs is very low.

**Table 4:** Availability of nano-pharmaceutical drugs in Palestinian hospitals

No.	Nano-drug	Total	Government	Private	NGOs	*P*-value
		*N* = 56 (%)	*N* = 29 (%)	*N* = 17 (%)	*N* = 10 (%)	
1	Ambisome®	17 (30.4)	8 (14.3)	6 (10.7)	3 (5.4)	0.860
2	Rapamune®	5 (8.9)	3 (5.4)	0 (0.0)	2 (3.6)	0.197
3	Ritalin La®	0 (0.0)	0 (0.0)	0 (0.0)	0 (0.0)	> 0.999
4	Emend®	3 (5.4)	0 (0.0)	0 (0.0)	3 (5.4)	0.001
5	Swiss Relief®	22 (39.3)	1 (1.8)	14 (25.0)	7 (12.5)	< 0.001
6	Invega®	1 (1.8)	0 (0.0)	1 (1.8)	0 (0.0)	0.311
7	Doxil®	2 (3.6)	2 (3.6)	0 (0.0)	0 (0.0)	0.381
8	Epaxal®	4 (7.1)	1 (1.8)	1 (1.8)	2 (3.6)	0.209
9	Mepact®	0 (0.0)	0 (0.0)	0 (0.0)	0 (0.0)	> 0.999
10	Mircera®	1 (1.8%)	1 (1.8)	0 (0.0)	0 (0.0)	0.623
11	Pegasys®	0 (0.0)	0 (0.0)	0 (0.0)	0 (0.0)	> 0.999
12	Somavert®	0 (0.0)	0 (0.0)	0 (0.0)	0 (0.0)	> 0.999

*P*-value > 0.05 is insignificant value

## Discussion

Pharmaceutical nanotechnology is a new branch of pharmaceutical sciences that creates novel nano-pharmaceutical drugs with improved therapeutic efficacy utilizing nanoscience. The number of available nano-pharmaceutical drugs has shown an exponential growth in the past decade. As this is a new science, there are no studies investigating the pharmacist’s knowledge of the available nano-pharmaceutical drugs and the availability of these drugs in the pharmaceutical market. Therefore, our study is the first worldwide to evaluate the knowledge among the Palestinian pharmacists of nano-pharmaceutical drugs and determine the availability of these drugs in the Palestinian hospitals.

Our results point out that the pharmacist’s knowledge of pharmaceutical nano-formulations is lower than that of conventional pharmaceutical formulations. The observed high pharmacist’s knowledge in conventional formulations is in agreement with previous studies that have also shown high pharmacist’s knowledge of conventional pharmaceutical compounding [35–37]. However, the low knowledge of nano-pharmaceutical drugs can be explained in two reasons; the development of nano-pharmaceutical drugs is a relatively new science that began in the late 2000s as shown in the report of the ISI web of knowledge by typing nanomedicine in the search engine of the website of ISI web of science database [38], and the other reason is the lack of related courses in the curriculum of pharmacy degree of most universities regarding pharmaceutical nanotechnology.

As noticed the knowledge of the Palestinian pharmacists in the pharmaceutical nano-formulations is not the same as their knowledge in the conventional pharmaceutical formulations. This indicates that they have low knowledge in this new field in Palestine.

In order to increase the awareness of this kind of drugs, there are some points that must be considered. The introduction of certain specialized courses in pharmaceutical nanotechnology would be very helpful, students should be educated about the advantages of this new technology as there is no program in bachelor years teaching pharmaceutical nanotechnology. Therefore, the pharmacy students will not have the sufficient knowledge of this interesting new field of therapy. Very recently in 2017, a pharmaceutical nanotechnology course has been introduced to the master program of pharmaceutical sciences at An Najah National University that provides a multidisciplinary graduate education course which combines the theoretical background and experimental application of nanoscience in the medical field.

Furthermore, we recommend establishing a series of workshops regarding pharmaceutical nanotechnology in order to increase the knowledge and the awareness of the graduated pharmacists about this new specialty that has huge impact in health field.

Moreover, it appears that the low availability for these nano-pharmaceutical drugs in the Palestinian hospitals isn’t the only problem in this field. There are many studies showing the lack of many other essential drugs, vaccines, and antidotes in the Palestinian hospitals [39, 40].

From the observed results, we conclude that some of the nano-pharmaceutical drugs are available in the Palestinian hospitals but with a very low frequency. The low availability could be explained due to their high price in comparison to the conventional available pharmaceutical formulations. As for example, the governmental hospitals purchase their drugs through vendor system which always prefers the lowest prices. Moreover, the low availability of nano-pharmaceutical drugs in Palestinian hospitals explains the obtained results of the low knowledge of these new pharmaceutical nano-formulations. Therefore, we recommend that the various health sectors should increase the availability of the FDA approved nano-pharmaceutical drugs in Palestine, at the time being there are more than 100 nano-pharmaceutical drugs that have been approved. Therefore, this will improve the patient’s health in Palestine.

### Strengths and limitations

The major strength of the current study is that it is the first of it’s kind to assess the availability and knowledge of pharmacists regarding nano-pharmaceutical drugs in Palestinian hospitals and worldwide.

This study is subject to a number of limitations. Our study was limited only to the Palestinian hospitals and didn’t include community pharmacies. Furthermore, the study findings were based entirely on a self-administered questionnaire survey. Four questions each for conventional pharmaceutical formulations and nano-formulations that may have not been sufficient to quantify the pharmacist’s knowledge. Finally, the data collected depend upon the knowledge and responsiveness of the respondents, which carries inherent risks of reporting error or bias.

## Conclusion

The questionnaire aimed to assess the knowledge and the availability of nano-pharmaceutical drugs in Palestinian hospitals. As the results of the nano-pharmaceutical drugs in governmental, private and NGO’s hospitals were only 8 out of 12 registered nano-pharmaceutical drugs available, and the frequency was very low, the conclusion of these results supports the hypothesis that pharmacist’s knowledge and the availability of nano-pharmaceutical drugs in Palestine is low.

## Additional file

Additional file 1: Questionnaire in English. (DOCX 39 kb)

## Abbreviations

FDA: Food and Drug Administration
IRB: Institutional review board
medic: Israeli medical index
NGOs: Non-governmental organizations
PMI: Palestinian medical index
