# Supplementary material for: A cross-sectional study of the availability and pharmacist’s knowledge of nano-pharmaceutical drugs in Palestinian hospitals
Source: BMC Health Serv Res. 2018 Apr 5;18:250. doi: 10.1186/s12913-018-3060-7 (PMC5887200; doi:10.1186/s12913-018-3060-7)
Supplement: Supplementary file 1 — Questionnaire in English. (DOCX 39 kb) [file 12913_2018_3060_MOESM1_ESM.docx]

**Supplementary I (English questionnaire)**


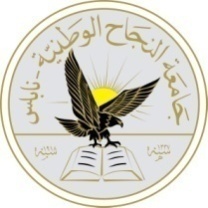


Department of Pharmacy

Faculty of Medicine & Health Sciences

An Najah National University

===================================================================

This questionnaire is only for the purposes of scientific research under the supervision of researchers from the pharmacy Department at the Faculty of Medicine and Health Sciences at An-Najah National University to evaluate the availability of nano-drugs and the extent of knowledge of pharmacist about the importance of this technology to improve drug development in various types of hospitals in Palestine..

You have full freedom and the will to participate in this research you have the right to take the time to consider whether or not to participate in the questionnaire and ask researcher what it deems appropriate and talk to any person or entity for this search. You can also inquire about the terms of any part in the research now or later, and if there are words or parts unintelligible. You can question the researcher and you'll have the time and adequate answer..

We emphasize that all of the information that will be collected are solely for the purposes of scientific research, and will be kept strictly confidential and not use this information for other purposes

. ===================================================================

Please fill in the following information:

*Date :..................................

*Job: ................................... * Experience Years :..................

* Graduation place : .................... *Educational level: ……………

*Hospital name :....................... *City................................

*Type Of Hospital :………………

- **Do the following pharmaceutical dosage forms are available in the hospital pharmacy and what is their distinctive properties:**

**(1) Enteric coated: Yes No**

**Choose the most characteristic feature of this pharmaceutical dosage form:**

a) Reduce the number of doses needed from the drug and achieve a slow/sustained release of the drug.

b) Aid in the protection of the drug from stomach acidic.

c) Used when the patient is susceptible to vomiting.

d) I don’t know.

**(2) Liposomes: Yes No**

**Choose the most characteristic feature of this pharmaceutical dosage form:**

a) Used when the patient is susceptible to vomiting

b) Aid in the protection of the drug from stomach acidity.

c) Reduce the side effects of the drug and improve its pharmacokinetic profile.

d) I don’t know.

**(3) Sublingual: Yes No**

- **Choose the most characteristic feature of this pharmaceutical dosage form:**

a) Reduce the number of doses needed from the drug and achieve a slow/sustained release of the drug.

b) Protect the drug from degradation or hydrolysis.

*c)* Fast drug absorption and protect the drug from hepatic metabolism.

d) I don’t know.

.

**(4) Nano emulsions: Yes No**

- **Choose the most characteristic feature of this pharmaceutical dosage form:**

a) Reduce the number of doses needed from the drug and achieve a slow/sustained release of the drug.

b) Aid in the protection of the drug from stomach acidity.

c) Used when the patient is susceptible to vomiting.

d) I don’t know.

**(5) Corticosteroid Inhaler: Yes No**

**Choose the most characteristic feature of this pharmaceutical dosage form:**

a) Allow the delivery of low water solubility drugs and permit the slow/sustained release of the drug.

b) The drug goes directly to the lungs which reduce the required dose and its side effects

c) Used when the patient is susceptible to vomiting

d) I don’t know.

**(6) Polymer nanoparticles: Yes No**

**Choose the most characteristic feature of this pharmaceutical dosage form:**

*a*) Protect the drug from degradation or hydrolysis

b) The drug goes directly to the lungs which reduces the required dose and its side effects.

c) Used when the patient is susceptible to vomiting.

d) I don’t know.

**(7) Suppositories: Yes No**

**Choose the most characteristic feature of this pharmaceutical dosage form:**

a) Protect the drug from degradation or hydrolysis

b) Reduce the number of doses needed from the drug and achieve a slow/sustained release of the drug

c) Used when the patient is susceptible to vomiting.

d) I don’t know.

**(8) Nano crystal dispersion: Yes No**

**Choose the most characteristic feature of this pharmaceutical dosage form:**

a) Aid in the protection of the drug from stomach acidity

b) Allow the delivery of low water solubility drugs and permit theslow/sustained release of the drug.

c) Used when the patient is susceptible to vomiting.

d) I don’t know.

- **Do the following medications available in any section of the hospital departments:**

|  | **Brand Name** | **Generic Name** | **available** | | **price**  **(NIS)** | **Not available** | **Monthly consumption** |
| --- | --- | --- | --- | --- | --- | --- | --- |
|  |  |  | **sometimes** | **always** |  |  |  |
| 1 | Ambisome | Amphotericin B |  |  |  |  |  |
| 2 | Rapamune | (Sirolimus) Rapamycin |  |  |  |  |  |
| 3 | Ritalin La | Methylphenidate Hydrochloride |  |  |  |  |  |
| 4 | Emend | Aprepitant |  |  |  |  |  |
| 5 | Swiss Relief | Diclofinac sodium |  |  |  |  |  |
| 6 | Invega | Paliperidone |  |  |  |  |  |
| 7 | Doxil | (Adriamycin) Doxorubicin |  |  |  |  |  |
| 8 | Epaxal | Hepatitis A vaccine |  |  |  |  |  |
| 9 | Mepact | Mifamurtide |  |  |  |  |  |
| 10 | Mircera | Epoetin Beta |  |  |  |  |  |
| 11 | Pegasys | Peginterferon alpha-2a |  |  |  |  |  |
| 12 | Somavert | Pegvisomant |  |  |  |  |  |

# 
